# Pharmacology Study of the Multiple Angiogenesis Inhibitor RC28-E on Anti-Fibrosis in a Chemically Induced Lung Injury Model

**DOI:** 10.3390/biom9110644

**Published:** 2019-10-24

**Authors:** Xiangying Kou, Yeying Sun, Shenjun Li, Weihua Bian, Zhihao Liu, Daolai Zhang, Jing Jiang

**Affiliations:** 1 Department of Pharmacology, Binzhou Medical University, Yantai 256603, China; xiangyingkou@sina.com (X.K.); sunyy21cn@163.com (Y.S.); bian_1005@163.com (W.B.); dlzhang313@163.com (D.Z.); 2 RemeGen Co., Ltd., Yantai 264006, China; lisj@remegen.cn (S.L.); liuzhihao@remegen.cn (Z.L.)

**Keywords:** VEGF, FGF, angiogenesis, anti-fibrosis, lung injury, pharmacology study

## Abstract

Background: Disease-related injury in any organ triggers a complex cascade of cellular and molecular responses that culminate in tissue fibrosis, inflammation, and angiogenesis simultaneously. Multiple cell angiogenesis is an essential part of the tissue damage response, which is involved in fibrosis development. RC28-E is a novel recombinant dual decoy receptor lgG1 Fc-fusion protein that can block vascular endothelial growth factor (VEGFA), platelet-derived growth factor (PDGF), and fibroblast growth factor-2 (FGF-2) simultaneously. This protein has stepped into clinical trials (NCT03777254) for the treatment of pathological neovascularization-related diseases. Here, we report on the role of RC28-E during anti-fibrosis and its potential multitarget function in regulating fibrosis. Methods: A bleomycin-induced pulmonary fibrosis C57BL/6 mouse model was established. Hematoxylin and eosin staining (HE) and Masson staining (Masson’s) were performed to evaluate the pulmonary fibrosis based on the scoring from, Ashcroft score. Fibrosis related factors and inflammatory cytokines including HYP, α-SMA, procollagen, ICAM, IL-6, IL-1, and TNF-α were also determined at the protein and mRNA levels to characterize the fibrosis. Both mRNA and protein levels of VEGF, FGF, and transforming growth factor (TGF)-β were detected by quantitative real-time PCR (qRT-PCR) and immunohistochemical (IHC) analysis, respectively. Pulmonary fibrosis and related cytokines were re-evaluated in vivo after 3 doses of RC28-E (5 mg/kg, 15 mg/kg, and 50 mg/kg, ip. Tiw × 9) in comparison with a mono-target antagonist treatment (VEGF or FGF blocking). RC28-E attenuated the activation of TGF-β induced fibroblasts in vitro. Expression levels of α-SMA and collagen I, as well as proliferation and migration, were determined with the human skin fibroblast cell line Detroit 551 and primary murine pulmonary fibroblast cells. The mechanism of RC28-E via the TGF-β/Smad pathway was also investigated. Results: RC28-E exhibits significant anti-fibrosis effects on Idiopathic pulmonary fibrosis (IPF) in vivo. Moreover, TGF-β induced fibroblast activation in vitro via the inhibition of the TGF-β downstream Smad pathway, thus providing potential therapeutics for clinical disease-related fibrosis-like IPF as well as chemotherapy-induced fibrosis in cancer therapy.

## 1. Introduction

Fibrosis and resulting organ failures account for at least one-third of the world’s death toll [1]. The repair response following tissue damage allows protecting the relative integrity of tissues and organs. However, when this response overreacts, it leads to organ fibrosis and loss of function [2]. This is mediated by multiple cellular and molecular elements including transforming growth factor (TGF-β), platelet-derived growth factor (PDGF), connective tissue growth factor (CTGF), vascular endothelial growth factor (VEGF), basic fibroblast growth factor (bFGF or FGF-2), and the integrin-extracellular matrix signaling pathway [3,4,5,6].

The VEGF and bFGF are potent pro-angiogenic factors that play an important role in tumor development. Previous studies have indicated cross-talk between the VEGF and FGF signaling pathways that appear to act synergistically in order to promote neovascularization [7,8,9]. The present study demonstrates that the expression of VEGF-A is up-regulated during liver fibrosis [10]. The bFGF or FGF-2, another multiple function factor, can enhance the proliferation, migration, and survival of endothelial cells [11] as well as tumor angiogenesis [12]. During fibrosis, fibroblast growth factor (FGF) also stimulates fibroblast migration to the injured site, increases collagen and fibronectin synthesis, inhibits the degradation of the extracellular matrix by metalloproteinases, and promotes the development of fibrosis [13]. Furthermore, this factor also promoted epithelial mesenchymal transition (EMT) by inducing the increased expression of a serial of factors like Vimentin, fibroblast specific protein-1 (FSP-1), α-smooth muscle actin (α-SMA), matrix metalloproteinase-2 (MMP-2), and matrix metalloproteinase-9 (MMP-9) and decreasing E-cadherin gene expression [14]. In another study, liver fibrosis resulting from chronic exposure to CCl4 was markedly decreased in the liver of FGF1/FGF2-deficient mice [15]. These results suggest that both VEGF and FGF play important roles during fibrosis. Several VEGF mono-targets and VEGF combined with other target therapies have been reported for fibrosis treatment. Ranibizumab (Lucentis^®^, Novartis) [16,17], one of the anti-VEGF therapeutics, approved by the FDA for the treatment of ocular fundus neovascularization disease, prevented epidural fibrosis in post laminectomy rat models [18]. Bevacizumab, a full-length humanized monoclonal antibody designed against all types of VEGF, was approved by the FDA in 2004 for the treatment of metastatic diseases and colon or rectal cancers [19]. Furthermore, this medicine was reported to hamper the activation of hepatocytes and the proliferation of hepatic stellate cells (HSCs) by blocking the effect of VEGF on HSCs, which could attenuate the development of hepatic fibrosis [20]. Although many clinical studies have deliberately described the significance of using anti-VEGF in fibrosis, the mechanism of action of anti-VFGF is not well understood [21,22].

**Figure 1:**
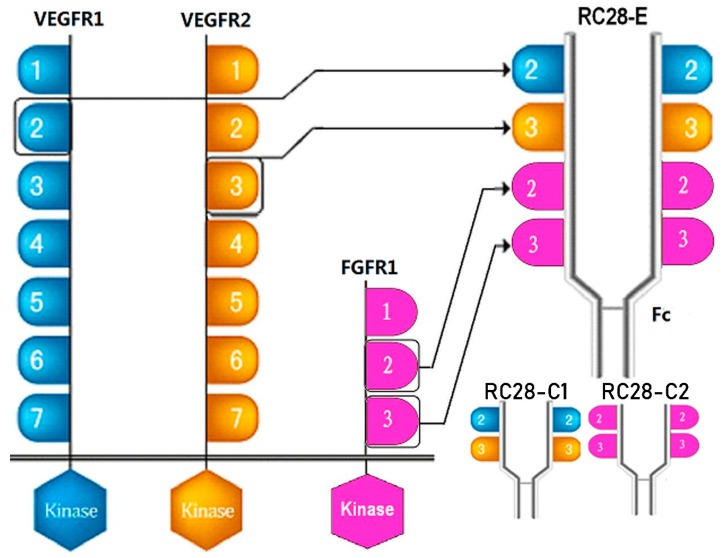
Schematic diagram of RC28 molecular design. VEGFR1: vascular endothelial growth factor receptor 1; FGFR1: fibroblast growth factor receptor 1.

TGF-β1 is a strong pro-fibrotic mediator that play multiple biological key roles like the epithelial mesenchymal transition (EMT), epithelial cell apoptosis, epithelial cell migration, and pro-fibrotic mediator production, etc. [23]. Recently, Park et al. reported the direct effect of VEGF on myofibroblast transformation via induction of TGF-β1 synthesis [24]. Furthermore, cross-talk between VFGF, FGF, and TGF-β during angiogenesis, tumorigenesis, and fibrosis have been reported [25,26,27]. Multiple target synergetic blocking strategy is a potential therapy for fibrosis-like idiopathic pulmonary fibrosis, chemotherapy induced pulmonary fibrosis, or hepatic fibrosis. RC28-E (as shown in Figure 1) is a novel recombinant dual decoy receptor lgG1 Fc-fusion protein that can inhibit VEGFA, FGF-2, and PDGF simultaneously [28]. RC28-E has been approved in clinical research (NCT03777254) for its therapeutic potency in angiogenesis-related diseases such as wet age-related macular degeneration, diabetic retinopathy, and multiple tumor cancers [29,30]. Given the evidence for the occurrence of both VEGF and FGF signaling in fibrogenesis, the purpose of the present study was to investigate the antifibrosis effect of RC28-E, especially in an idiopathic pulmonary fibrosis model, providing new insight and experimental basis for the treatment of fibrosis.

## 2. Materials and Methods

### 2.1. Drugs

RC28-E, a recombinant VEGF and FGF receptor lgG1 Fc-fusion protein (RemeGen, Ltd., Yantai, Shandong, China), was used for animal and cell administration. VEGF-trap (Lot# RC28-C1-20180124) and FGF-trap (Lot# RC28-C2-20180527) were obtained from RemeGen, Ltd.

### 2.2. Animals

Male C57BL/6 mice of 6–8 weeks old, and weighing 20–25 g, were purchased from Jinan Pengyue Experimental Animal Breeding Co., Ltd. (Shandong, China). Animals were randomly sub-caged in specific pathogen free animal laboratory (SPF)-grade environments. All animal procedures were approved by the Institutional Animal Care and Use Committee of Binzhou Medical University and were performed in accordance with the National Research Council (US) Committee Guide for the Care and Use of Laboratory Animals. Animals were housed in metal breeding cages with free access to food and water in a room with a 12-h/12-h light/dark cycle. The humidity and temperature were maintained at 55% and 23 °C, respectively. All reasonable efforts were made to minimize suffering.

### 2.3. Bleomycin-Induced Mouse Pulmonary Fibrosis Model and Treatment

C57BL/6 male mice were randomly divided into 7 groups (n = 7): the control group (n = 7), model group (n = 7), RC28-E H treatment group (50 mg/kg, n = 7), RC28-E M treatment group (15 mg/kg, n = 7), RC28-E L treatment group (5 mg/kg, n = 7), RC28-C1 treatment group (35 mg/kg, n = 7), and RC28-C2 treatment group (35 mg/kg, n = 7). Mice were placed in an individual ventilated cages (IVC) closed cage under awake state, and 0.25% bleomycin was atomized with the use of a nebulizer into the cage through a nebulizing tube 6 times for 30 min each time. Mice of the control group were treated with the same amount of saline buffer prior to mice erection and rotation to ensure that the drug distributed evenly into the lungs. On the second day of modeling, the control and model groups were intraperitoneally injected with physiological saline buffer (20 mL/kg) three times a week for 3 weeks. Mice of the RC28-E H, RC28-E M, RC28-E L, RC28-C1, and RC28-C2 groups were injected intraperitoneally with 50, 15, 5, 35, and 35 mg/kg, respectively, three times a week for 3 weeks. At the end of this period, mice were all sacrificed by intraperitoneal injection with 1% pentobarbital sodium (40 mg/kg) on the 21st day after modeling, and lung tissue were removed. The thoracic cavity was opened, and the right hilum was ligated. The left lung was perfused. The upper lobe of the right lung was fixed in neutral buffered formaldehyde, embedded into paraffin, and sectioned before HE and Masson staining. The right lower lobe of the lung was kept in liquid nitrogen and transferred to −80 °C for storage.

### 2.4. Isolation of Primary Murine Fibroblasts

Male C57BL/6 mice of 6–8 weeks old and weighing 20–25 g were used in this study. The extracted pulmonary was washed with Dulbecco’s phosphate-buffered saline (DPBS) solution (pH 7.4, Gibco BRL, Grand Island, NY, USA) without Ca^2+^ and Mg^2+^. The tissues were sliced into approximately 1 mm^3^ pieces with micro-dissecting scissors, and treated with 10 mL of collagenase II (0.8 mg/mL, 262 units/mg, Gibco BRL, Grand Island, NY, USA) for 5 min at room temperature. The initial supernatant was discarded by decantation and the remaining tissues were treated with fresh collagenase II solution for an additional 5 min at 37 °C. The supernatant containing cells were transferred into a tube prefilled with cell culture medium (MEM containing 10% fetal bovine serum, Gibco BRL, Grand Island, NY, USA), and centrifuged at 1200 rpm for 4 min at room temperature. The cell pellets were re-suspended in 5 mL of cell culture medium. The above procedures were repeated 7–9 times until little tissues were left. The cell suspensions were collected and incubated in 100 mm tissue culture dishes for 30 min for attachment. Unattached cells were then discarded by replacing the culture medium. Attached fibroblasts were subsequently cultivated in MEM containing 10% (*v*/*v*) FBS in a CO_2_ incubator at 37 °C. This cell line was used for further experiments upon reaching 70–80%.

### 2.5. Cell Culture

The human skin fibroblast cell line Detroit 551 was purchased from AllCells (Shanghai, China) and maintained in MEM medium (Gibco, Grand Island, NY, USA) supplemented with 10% fetal bovine serum, 1% penicillin, and streptomycin under 5% CO_2_ atmosphere at 37 °C. This cell line was used for further experiments upon reaching 70–80%.

### 2.6. Histopathological Analysis

The upper lobe of the right lung was fixed in 10% buffered formalin prior to paraffin embedding. Sections were stained with hematoxylin, eosin, and Masson staining. The fibrotic states were quantified based on the study from Ashcroft score [31]. The mean score was considered as the fibrotic score.

### 2.7. Detecting Hydroxyproline (HYP) in Lung Tissues by Alkaline Hydrolysis

A quantity of 40–100 mg of lung tissues was placed into a test tube. Tissues were mixed with 2 mL of hydrolysase and placed into a boiling water bath for 1 h. The supernatant was centrifuged at 16,000 rpm/min for 20 min according to the instructions of the manufacturer (HYP detection kit, Solarbio, Beijing, China). The absorbance at 560 nm was measured with a spectrophotometer, and the HYP content of each lung tissue sample was calculated.

### 2.8. Immunohistochemistry

Four micrometer-thick slides were deparaffinized with xylene before rehydration using an ethanol gradient. Quenching of the endogenous peroxidase activity was achieved by incubating samples with endogenous peroxidase blocker. After blocking with 3% BSA, sections were incubated with mouse anti-α-SMA (Cell Signaling Technology, Boston, MA, USA), mouse anti-TGF-β (R&D, Minneapolis, MN, USA), rabbit anti-VEGFA (Abcam, Cambridge, UK), and mouse anti-FGF basic/FGF2 (Novus, Littleton, CO, USA) overnight at 4 °C. Horseradish peroxidase-conjugated goat anti-mouse/rabbit lgG was used as the secondary antibody. The DAB Substrate System (DAKO) was used to reveal the immunohistochemistry staining.

### 2.9. Cytometric Bead Analysis

Levels of the cytokines IL-6, IL-1, and TNF-α were measured simultaneously by cytometric bead array using the mouse IL-6/ IL-1/TNF-α Cytokine Kit (R&D, Systems, Minneapolis, MN, USA) according to the manufacturer’s instructions.

### 2.10. RNA Extraction and Quantitative Real-Time Polymerase Chain Reaction (Q-PCR)

Total RNA was extracted from cultivated cells or lung tissues using Trizol reagent (Takara, Shiga, Japan). Approximately 1 mL of Trizol was directly added to each sample. Each sample was then incubated at room temperature for 30 min prior to the addition of 200 μL of chloroform. Samples were incubated for 15 min at room temperature with shaking and subsequently centrifuged at 12,000 rpm at 4 °C for 15 min. The samples were divided into three layers. The aqueous phase was reserved with 500 μL of isopropyl alcohol at room temperature for 15 min. Samples were centrifuged at 10,000 rpm for 10 min at 4 °C and the supernatant was removed. The RNA pellet was gently washed using 75% (*v*/*v*) ethanol. The samples were then centrifuged at 7500 rpm at 4 °C for 10 min, and the supernatant was discarded. The pellet was air-dried at room temperature and dissolved in 20 μL of RNase-free water. First-strand cDNAs were synthesized using the PrimeScript™ RT reagent Kit with gDNA Eraser (Perfect Real Time) (Takara, Shiga, Japan). Quantitative RT-PCR was performed with the StepOnePlus Real-Time PCR System (Thermo Fisher Scientific). All samples were run in duplicate and the results were averaged and normalized to the expression level of GAPDH (glyceraldehyde-3-phosphate dehydrogenase). The CT value (amplification power curve inflection point) was obtained, ∆Ct = CT (target gene) − CT (internal reference), ∆∆Ct = ∆Ct (treatment group) − ∆Ct (control group); the relative expression of target genes was calculated using 2^−∆∆Ct^.

### 2.11. Western Blot Analysis

Protein expression levels were measured by western blot analysis. Cells were lysed with RIPA lysis buffer (Beyotime, Shanghai, China). Protein concentrations were determined using the BCA protein assay kit (Beyotime, Shanghai, China). Sample buffer was added to reach 30 μg of sample per well. Proteins were boiled at 95 °C for 5 min. Proteins were then separated by 10% SDS–PAGE and transferred to polyvinylidene fluoride (PVDF) membranes. Samples were subsequently incubated at room temperature for 2 h with 5% skimmed milk prior to the incubation with the first antibody (1:1000 dilution) overnight at 4 °C. The membrane was washed three times with 0.05% TBS-T for 5 min and incubated at room temperature with the horseradish peroxidase-conjugated secondary antibody for 2 h. Immune-positive bands were detected with the use of the enhanced chemiluminescence (ECL) reagent (GE Healthcare, UK). Band intensities were quantified using Image J.

### 2.12. Immunofluorescence Staining

Cell suspensions with a density of 2.5 × 10^5^ were plated on sterile slides in 24-well plates and cultured in serum-free MEM medium for 24 h. Drugs were added 30 min before the addition of TGF-β1 (R&D, Systems, Minneapolis, MN, USA). Cells were then washed with PBS three times and fixed with 4% paraformaldehyde in PBS at room temperature for 30 min. The cells were subsequently washed with PBS three times, incubated with 0.5% Triton X-100 for 15 min at room temperature, and blocked with 1% BSA for 1 h. Samples were incubated with antibodies specific for α-SMA (1:100, R&D, MN, USA) overnight at 4 °C. Then, samples were washed with PBS three times and incubated with goat anti-mouse IgG Fc (DyLight^®^ 594) (Abcam, Cambridge, UK) at room temperature for 1 h. After rinsing three times with PBS, the nuclei were stained with DAPI (Beyotime, Shanghai, china) for 10 min at room temperature. Immunofluorescence was analyzed under a fluorescence microscope.

### 2.13. Cell Proliferation Assay

In order to evaluate the capacity of RC28-E to inhibit cell proliferation, cells (5 × 10^3^ cell/well) were seeded in 96-well plates and cultured in basic medium containing 1% fetal bovine serum (FBS) overnight. Subsequently, samples were treated with RC28-E for 30 min before the addition of TGF-β1 (R&D, Systems, Minneapolis, MN, USA). When the treatment was completed, 10% Cell Counting Kit-8 (Dojindo Molecular Technologies, MD, USA) was added to each well. The plate was continuously incubated for 3 h. Finally, the absorbance was measured with a microplate reader at 450 nm.

### 2.14. Transwell Migration Assay

In order to assess the cell migration capacity, cells were plated in the upper chamber of Transwell assay plates with 8 μm filter pore size (Costar Corning, Corning, NY, USA). The cells were incubated in serum deprivation media for 12 h, and the drug was added to the upper chamber. After 1 h, TGF-β1 was added to the bottom chamber, and incubated at 37 °C for 24 h. Non migrated cells were removed, and migrated cells were fixed with 4% polymethanol for 30 min following incubation with crystal violet for 30 min in the dark. The cells were quantified by manual counting.

### 2.15. Statistical Analysis

Data were presented as means ± SD (X ± SD) and analyzed with GraphPad Prism (GraphPad Software, San Diego, CA, USA). The difference between the mean of the groups was determined by ANOVA. Unpaired two-tailed *t*-test was used to investigate the differences between the groups. *p* < 0.05 was considered statistically significant.

## 3. Results

### 3.1. Bleomycin-Induced Pulmonary Fibrosis Formation

**Figure 2:**
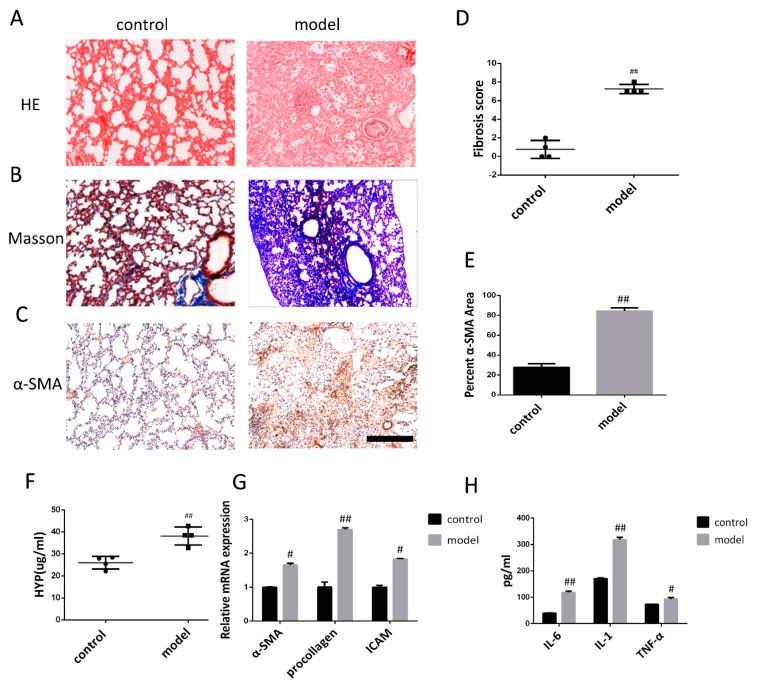
Pathological changes in lung tissue and the level of inflammatory cytokine in model of mice after bleomycin-induced pulmonary fibrosis formation. (**A**) The lung section from the mice that have undergone different treatments were stained by hematoxylin–eosin (HE) (n = 4 per group). (**B**) Lung sections from various treatment groups were subjected to Masson trichrome staining (n = 4 per group). (**C**) The expression of α-SMA was determined by using immunohistochemical analysis. Scale bar = 200 μm for each picture (original magnification: ×100, n = 4 per group). (**D**) The lung fibrosis score based on the scoring from Ashcroft score. (**E**) Quantitative analysis of α-SMA positive area. (**F**) Hydroxyproline concentration in lung tissue was detected by using alkaline hydrolysis (n = 4 per group). (**G**) Change of α-SMA, procollagen, and ICAM transcripts were analyzed by qRT-PCR (n = 6 per group). (**H**) The expression of IL-6, IL-1, and TNF-α was examined by using ELISA in the lung and serum. All data are expressed as the mean ± SD. ^#^ *p* < 0.05, ^##^ *p* < 0.01 versus control.

Three weeks after modeling, hematoxylin and eosin staining (HE) and Masson staining (Masson’s) were carried out in experimentally stain control and bleomycin-induced (BLM) mice to examine the formation of pulmonary fibrosis (Figure 2A,B). In the control group, the alveolar structure of HE staining was normal, the lung tissue was well structured, and there was no inflammatory cell infiltration. Masson staining revealed a small amount of blue-stained collagen fibers in the peribronchial and alveolar spaces. The bleomycin induced model (BLM) showed obvious alveolar inflammatory changes, HE stained alveolar septal edema thickening, structural destruction, massive inflammatory cell infiltration in the alveolar space, interstitial macrophages and neutrophils, as well as a small number of lymphocytes. Moreover, Masson staining showed collagen fibrosis. The difference between control and model groups was statistically significant. Furthermore, α-SMA immunohistochemical analysis showed that alveolar epithelial cells in the lung tissue of the control group were not stained, while alveolar epithelial cells in the lung tissue of the bleomycin induced model (BLM) were stained (Figure 2C). The fibrosis scores were significantly lower than the bleomycin induced model (BLM) (Figure 2D), indicating that pulmonary fibrosis was installed three weeks after modeling.

Alkaline hydrolysis was used to evaluate the hydroxyproline concentration, which was significantly higher in the lung tissue homogenate from the bleomycin induced model (BLM) than in the control group (Figure 2F). The mRNA levels of α-SMA, procollagen, and ICAM detected by qRT-PCR exhibited increased after bleomycin treatment compared to the saline treated control group (Figure 2G). ELISA analysis revealed that the levels of IL-6, IL-1, and TNF-α in bleomycin-treated animals were significantly higher compared to the control (Figure 2H).

### 3.2. Increased VEGF, FGF, and TGF-β Expression in the Lung Following Bleomycin-Induced Pulmonary Fibrosis Formation

**Figure 3:**
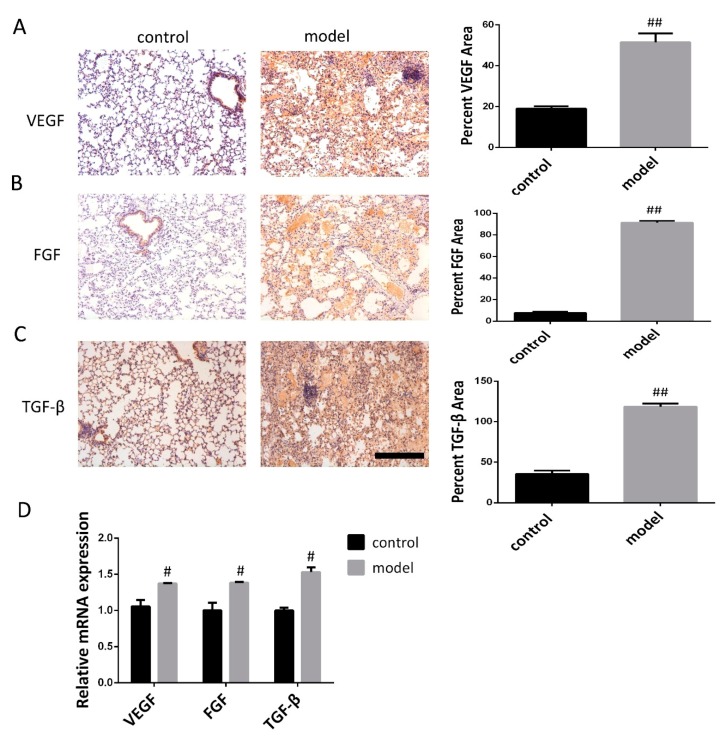
Expression levels of VEGF, FGF, and TGF-β in lung of mice after bleomycin-induced pulmonary fibrosis formation. (**A**–**C**) The VEGF, FGF, and TGF-β expression levels in the control mice and modeling mice were determined by immunohistochemistry assay. Scale bar = 200 μm for each picture (original magnification: X100, n = 4 per group). Quantitative analysis of VEGF, FGF, and TGF-β positive area. Densitometry data are shown as mean ± SD. (**D**) The levels of VEGF, FGF, and TGF-β mRNA in lung tissue were detected by qRT-PCR (n = 6 per group). Results are shown as mean ± SD. ^#^ *p* < 0.05, ^##^ *p* < 0.01 versus control.

Three weeks after bleomycin induced pulmonary fibrosis formation, immunohistochemical analysis and qRT-PCR were carried out in experimentally control and experimental (bleomycin) mice to examine relative protein and mRNA expression. Immunohistochemical analysis showed that VEGF, FGF, and TGF-β were weakly stained in control mice, while VEGF, FGF, and TGF-β protein expression remained at a high level in the bleomycin induced model (BLM) (Figure 3A–C). Furthermore, the expression levels of VEGF, FGF, and TGF-β mRNA were determined by qRT-PCR during the development of bleomycin-induced pulmonary fibrosis; we also found that mRNA levels of VEGF, FGF, and TGF-β were increased in the modeling group but decreased in the control group (Figure 3D). This revealed a significantly increased expression of VEGF, FGF, and TGF-β following bleomycin-induced pulmonary fibrosis formation when compared with controls.

### 3.3. Effects of RC28-E on the Expression of VEGF, FGF, and TGF-β During Bleomycin-Induced Fibrosis Formation

**Figure 4:**
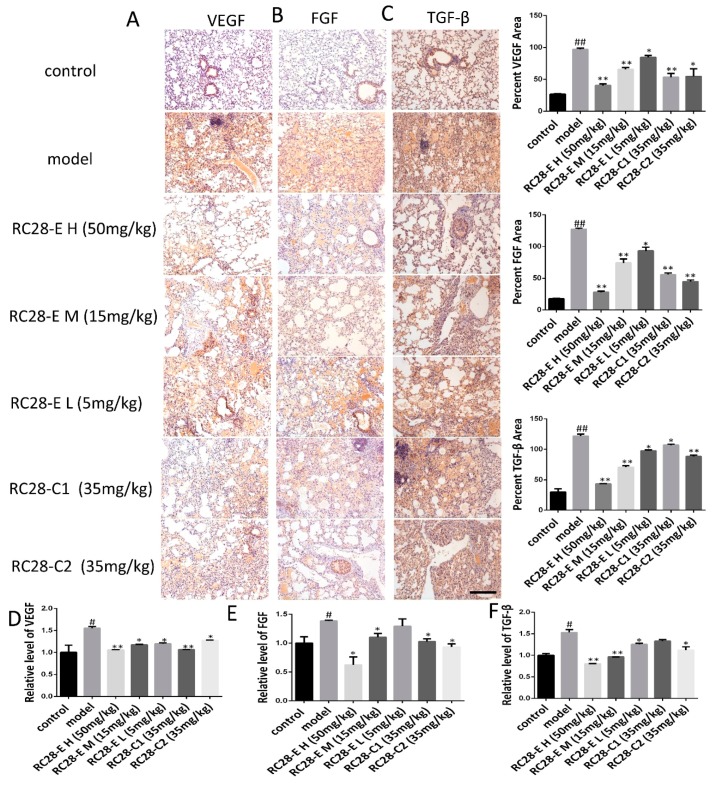
Effect of RC28-E on VEGF, FGF, and TGF-β expression in bleomycin-induced fibrosis formation. (**A**–**C**) Immunohistochemistry was used to examine VEGF, FGF, and TGF-β expression in lung tissues. Scale bar = 200 μm for each picture (original magnification: ×100). (**D**–**F**) The change of VEGF, FGF, and TGF-β transcripts were analyzed by qRT-PCR. (n = 4 per group). Results are shown as mean ± SD. * *p* < 0.05, ** *p* < 0.01 versus model alone. ^#^ *p* < 0.05, ^##^ *p* < 0.01 versus control.

The TGF-β protein expression level is elevated in the bleomycin induced model (BLM) and may promote the occurrence of fibrosis by regulating the expression of VEGF and FGF [27]. To elucidate whether RC28-E affects VEGF, FGF, and TGF-β related to fibrosis formation, the effect of RC28-E on VEGF, FGF, and TGF-β protein expression levels were determined in lung during fibrosis formation. As shown in Figure 4A–C, the expression of VEGF, FGF, and TGF-β was detected by immunohistochemistry in these lung tissues. Indeed, the results showed that the expression of VEGF, FGF, and TGF-β was null in control lung tissues while they were up-regulated during pulmonary fibrosis. Moreover, compared with bleomycin induced model, administration of RC28-E induced a significant down-regulation of VEGF, FGF, and TGF-β in fibrosis lungs in a concentration-dependent manner, and better suppression of RC28-E than a mono-target antagonist treatment (RC28-C1 or RC28-C2). Similarly, as shown in Figure 4D–F, the mRNA expression of VEGF, FGF, and TGF-β was examined by qRT-PCR. Bleomycin-induced up-regulation of VEGF, FGF, and TGF-β were inhibited by RC28-E in a concentration-dependent manner. Moreover, RC28-E showed a better inhibitory effect than RC28-C1 (VEGF-trap) and RC28-C2 (FGF-trap) at the same concentration. These results suggest that lung epithelial cells might produce VEGF and FGF during the formation of pulmonary fibrosis. RC28-E improves fibrosis by targeting VEGF-R and FGF-R.

### 3.4. Effects of RC28-E on Lung Fibrosis Induced by Bleomycin in Mice

**Figure 5:**
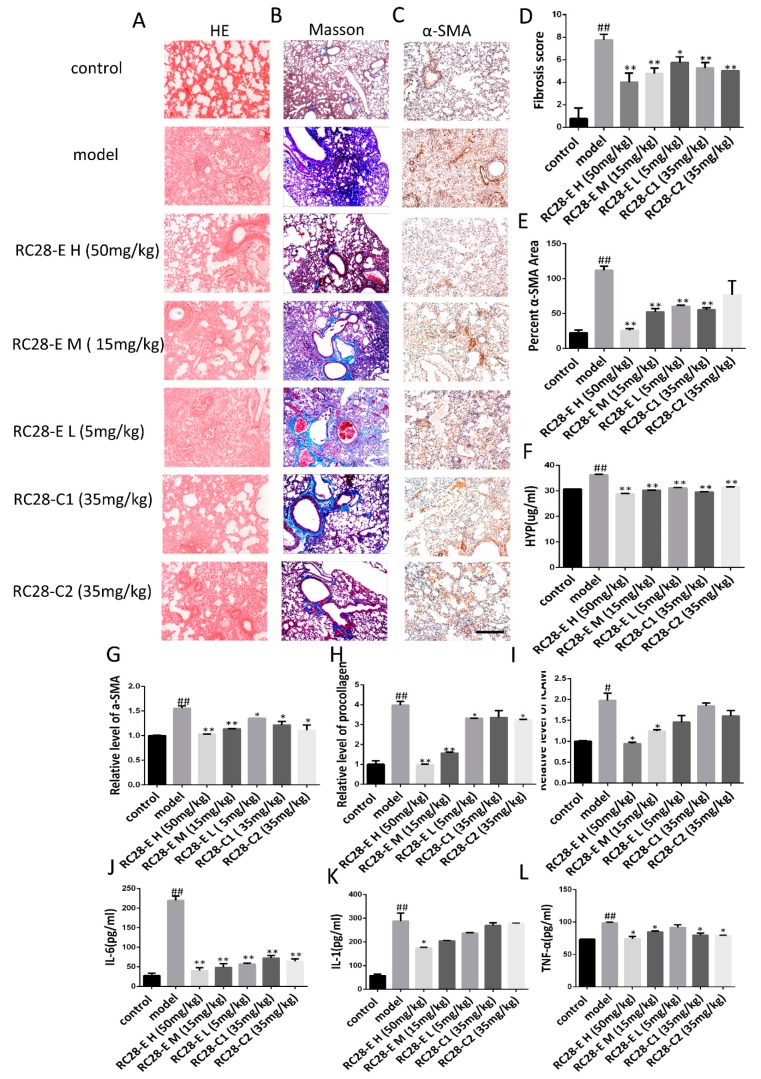
The effect of RC28-E on the development of fibrosis in a therapeutic mouse bleomycin model. (**A**) The lung sections from the mice that have undergone different treatments were stained by hematoxylin–eosin (HE). (**B**) Lung sections from various treatment groups were subjected to Masson trichrome staining. (**C**) Immunohistochemistry was used to examine α-SMA expression in pulmonary tissues. Scale bar = 200 μm for each picture (original magnification: ×100, n = 4 per group). (**D**) The lung fibrosis score based on the scoring from Ashcroft score. (**E**) Quantitative analysis of α-SMA positive area. (**F**) The effect of RC28-E on hydroxyproline concentration in lung tissue. (**G**–**I**) The expression of α-SMA, procollagen, and ICAM mRNA were detected by qRT-PCR. (**J**–**L**) IL-1, IL-6, and TNF-α ELISA kit were used to detect the IL-1, IL-6, and TNF-α expression in the bleomycin induced lung tissue. Results are shown as mean ± SD. * *p* < 0.05, ** *p* < 0.01 versus model alone. ^#^ *p* < 0.05, ^##^ *p* < 0.01 versus control.

HE and Masson’s trichrome staining were used for assessing the histopathology of lung tissues. As shown in 5A, HE staining showed control lung tissue structure and intact alveolar cavities with few inflammatory cells in the control group. In the model group, significant infiltration of inflammatory cells in the alveolar space were observed, as well as alveolar structure disorder. The alveolar septum was significantly widened and the alveolar wall thickened. Fibroblast proliferation and pulmonary fibrosis scar formation were also observed in the modeling group. In RC28-E treated groups, the alveolar septum was less thickened and inflammatory cell infiltration decreased in a concentration-dependent manner compared to the modeling group. Additionally, the potency of the inhibiting effect for RC28-C1 (VEGF-trap) and RC28-C2 (FGF-trap) were similar to that of RC28-E on histopathology of lung tissues, while the maximum relative anti-inflammatory effect of RC28-E H (50 mg/mL) was significantly superior to the other mono-target antagonist treatment (RC28-C1 or RC28-C2). As shown in Figure 5B, Masson’s trichrome staining was used to observed fibrosis in lung tissues. In control lungs, a thin blue color of collagen was observed, whereas excessive collagen deposition, inflammatory cell infiltration, and a damaged lung tissue structure were visible in model lung tissues. Compared with the modeling group, the alveolar wall thickness and inflammatory cell infiltration exhibited a concentration-dependent manner in RC28-E treated groups. Furthermore, RC28-E exhibited a better therapeutic effect of bleomycin induced fibrosis as compared to RC28-C1 (VEGF-trap), and similar inhibition of fibrosis to RC28-C2 (FGF-trap). In addition, pulmonary tissue damage was decreased and only a small amount of collagen fiber deposition could be observed in RC28-E treated groups. As presented in Figure 5C, bleomycin injury denuded the alveolar airspaces accompanied by extensive α-SMA expression in the bleomycin induced model, while treatment with RC28 could improve the lung structure, reduce interstitial fibrosis, and decrease the protein expression of α-SMA. Moreover, compared with the group that mono-targeted antagonist treatment (RC28-C1 or RC28-C2), administration of RC28-E resulted in profound decreases in the protein expression of α-SMA at the same concentration. On the other hand, HE and Masson‘s were performed to evaluate the pulmonary fibrosis based on the scoring from the Ashcroft score. As shown in Figure 5D, RC28-E exhibited a better amendable effect on fibrosis compared to RC28-C1 (VEGF-trap) and RC28-C2 (FGF-trap) at the same concentration.

The hydroxyproline (Hyp) content is an important indicator reflecting the degree of collagen tissue metabolism and fibrosis. We thus assessed the effects of RC28-E on the hydroxyproline concentration during bleomycin-induced lung fibrosis. As shown in Figure 5F, we found that the hydroxyproline concentration was significantly higher in lung tissues of the model group compared to the control group. RC28-E effectively decreased the hydroxyproline concentration in a concentration-dependent manner. However, these effects were more pronounced with RC28-E H (50 mg/mL) than RC28-C1 (VEGF-trap) and RC28-C2 (FGF-trap). Similarly, as shown in Figure 5G–I, the mRNA expression of the profibrogenic gene was examined by qRT-PCR. Bleomycin-induced up-regulation levels of α-SMA, procollagen, and ICAM were decreased by RC28-E in a concentration-dependent manner. Furthermore, RC28-E showed a preferable treatment effect compared to RC28-C1 (VEGF-trap) and RC28-C2 (FGF-trap) at the same concentration.

Acute and chronic inflammation often triggers fibrosis. Inflammation leads to injury of resident epithelial cells and often endothelial cells, resulting in the enhanced release of inflammatory mediators, including cytokines, chemokines, and others. This process leads to the recruitment of a wide range of inflammatory cells, which elicit the activation of effector cells and drive the fibrogenic process. We then investigated whether VEGF/FGF inhibition could inhibit inflammation and the subsequent lung fibrosis in vivo. As shown in Figure 5J–L, the levels of IL-1, IL-6, and TNF-α in the bleomycin-induced mouse pulmonary fibrosis model were higher than those in the control group, while RC28-E treated mice showed a significant reduction in the levels of the proinflammatory cytokines IL-1, IL-6, and TNF-α in a concentration-dependent manner. Similarly, our data showed that RC28-C1 (VEGF-trap) and RC28-C2 (FGF-trap) were also able to reduce the levels of proinflammatory cytokines as compared to the modeling group. Nevertheless, these results suggest that RC28-E is more effective than RC28-C1 (VEGF-trap) and RC28-C2 (FGF-trap) on the levels of proinflammatory cytokines at the same concentration. Furthermore, high RC28-E and RC28-C don’t affect control animals (Figure S1).

### 3.5. RC28-E Inhibits TGF-β1-Induced Fibroblast Activation In Vitro

**Figure 6:**
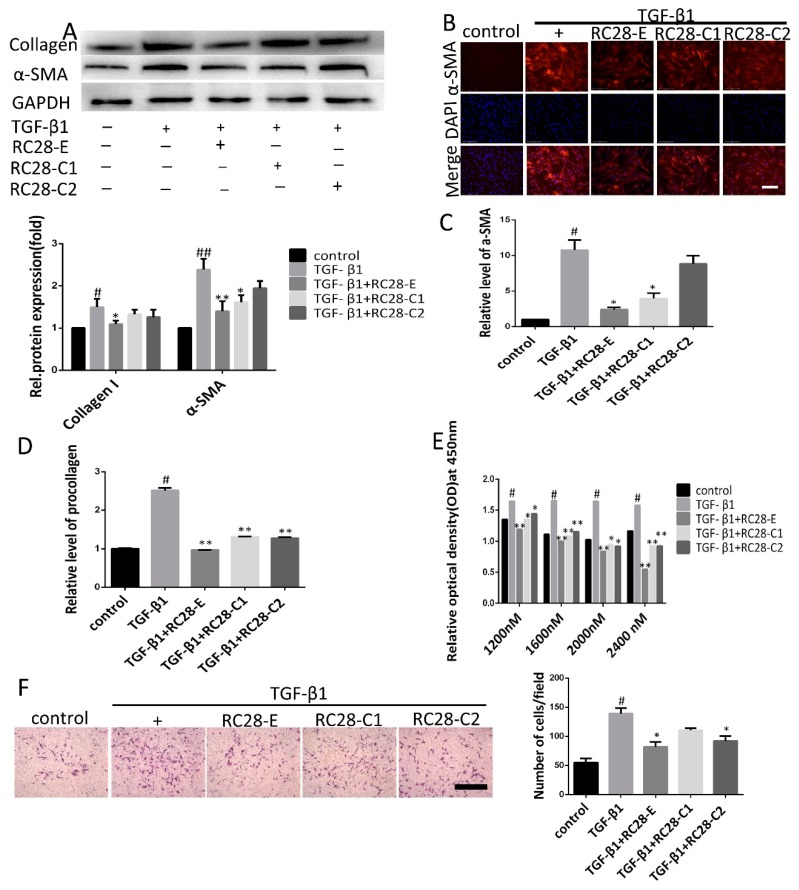
RC28-E attenuates TGF-β-induced fibroblast activation. (**A**–**D**) Human skin fibroblasts line Detroit 551 were treated with RC28-E (2400 nM) or 2400 nM RC28-C1 (VEGF-trap) and RC28-C2 (FGF-trap) in the presence hTGF-β1 (10 ng/mL) for 72 h. (**A**) The protein expression levels of α-SMA and collagen I were examined by western blot and GAPDH as loading control. Quantitative data were from western blot analysis (n = 3). (**B**) The expression of α-SMA was detected by immunofluorescence assay. Nuclei were stained with DAPI (blue). Scale bar = 275 μm for each picture (original magnification: ×200). (**C**,**D**) Changes of α-SMA and procollagen transcripts were analyzed by qRT-PCR. (**E**) cells were treated with 1200, 1600, 2000, and 2400 nM RC28-E or 1200, 1600, 2000, and 2400 nM RC28-C1 (VEGF-trap) or 1200, 1600, 2000, and 2400 nM RC28-C2 (FGF-trap), with hTGF-β1 (10 ng/mL) as the control, for 48 h. Relative cell proliferation was determined by using cell counting kit-8. (**F**) Transwell was used to migration analysis, the migrated cells were stained with crystal violet. Results are shown as mean ± SD. * *p* < 0.05, ** *p* < 0.01 versus hTGF-β1 alone. ^#^ *p* < 0.05, ^##^ *p* < 0.05 versus control (n = 3).

Previous studies have reported that TGF-β1 promotes the transformation of fibroblasts into myofibroblasts, stimulates the synthesis of extracellular matrix proteins, and inhibits their degradation [32]. We further examined whether RC28-E inhibition of fibroblast activation could mitigate fibrogenic responses of both human and mouse fibroblasts. Fibroblasts were treated with TGF-β1 (10 ng/mL) for 72 h in the absence or presence of RC28-E. As presented in Figure 6A, cells treated for 72 h with hTGF-β1 displayed a robust expression for α-SMA, suggesting that differentiation into myofibroblasts had taken place. In contrast, hTGF-β1 induced expression of α-SMA and collagen I in the human skin fibroblast line Detroit 551 were effectively inhibited by RC28-E and mono-target antagonist treatment (RC28-C1 or RC28-C2). Immunofluorescence staining confirmed that both RC28-E and mono-target antagonist treatment (RC28-C1 or RC28-C2) were able to suppress hTGF-β1-mediated expression of α-SMA in the human skin fibroblast line Detroit 551 (Figure 6B). Comparable results were obtained when α-SMA and collagen I were analyzed at the transcription level (Figure 6C,D). These results showed that RC28-E significantly decreased the expression of both α-SMA and collagen I, indicating that TGF-β1-induced fibroblast activation was significantly attenuated by RC28-E, while these effects were more pronounced with RC28-E compared to RC28-C1 (VEGF-trap) and RC28-C2 (FGF-trap). We identified fibroblasts by FSP1 (fibroblast specific protein 1) and EpCAM (epithelial cell adhesion molecule) using flow cytometry. For FSP1, a specific marker for fibroblasts, isolated fibroblasts were positive, but negative for EpCAM, a marker of epithelial cell (Figure S2). We also confirmed that RC28-E suppressed the expression of the mTGF-β1-induced myofibroblast marker in primary mouse fibroblasts, which was consistent with the findings in human skin fibroblasts (Figure S3A–D).

To assess the effects of RC28-E on fibroblast proliferation, the viability of human skin fibroblasts submitted to RC28-E treatment in the presence of hTGF-β1 (10 ng/mL) was determined using the cell counting kit-8 (CCK-8). Our results demonstrated that the proliferation of the human skin fibroblast line Detroit 551 was promoted by hTGF-β1 (10 ng/mL) while it was significantly inhibited by RC28-E in a concentration-dependent manner. Moreover, compared with RC28-C1 (VEGF-trap) and RC28-C2 (FGF-trap), RC28-E enhanced the cell proliferation inhibition effect at the same concentration (Figure 6E). To investigate the effect of RC28-E on cell migration, we performed a Transwell assay. This analysis showed that RC28-E exhibited a higher inhibition of TGF-β1-induced migration as compared to RC28-C1 (VEGF-trap) and RC28-C2 (FGF-trap) at the same concentration (Figure 6F). We also confirmed that RC28-E suppressed the proliferation of mTGF-β1-induced fibroblasts and the migration in primary mouse fibroblasts, which was consistent with our findings in human skin fibroblasts (Figure S3E,F).

### 3.6. RC28-E Downregulated Protein Levels of VEGF and FGF and Inhibited the Phosphorylation Activity of the Downstream Signaling Molecules Smad in Human Skin Fibroblasts

**Figure 7:**
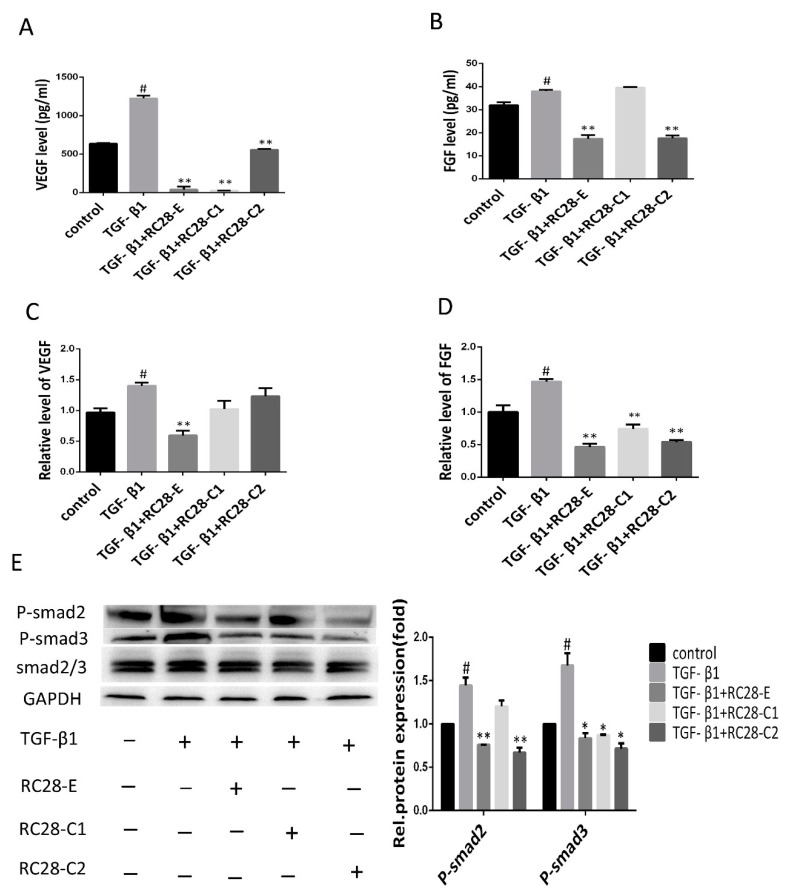
RC28-E downregulated protein levels of VEGF and FGF and inhibited the phosphorylation activity of the downstream signaling molecules Smad in human skin fibroblasts. (**A**–**E**) Human skin fibroblasts Detroit 551 were pretreated with RC28-E (2400 nM) for 1 h, followed by the addition of hTGF-β1 into the culture medium to a final concentration of 10 ng/mL and incubation for 72 h. (**A**,**B**) The protein levels of VEGF and FGF were examined by ELISA. (**C**,**D**) The expression of VEGF and FGF mRNA were analyzed by qRT-PCR. (**E**) The expression of P-smad2, P-smad3, and smad2/3 was determined using western blot and GAPDH as loading control. Representative gel electrophoresis bands are shown, and the expression levels of proteins were quantified by densitometry and normalized to the expression of smad2/3. Densitometry data are shown as mean ± SD. * *p* < 0.05, ** *p* < 0.01 versus hTGF-β1 alone. ^#^ *p* < 0.05, ^##^ *p* < 0.05 versus control (n = 3).

To investigate the molecular mechanism of RC28-E in the inhibition of cell growth and fibrosis control, an enzyme-linked immunoassay (ELISA) and qRT-PCR were used to determine the expression of VEGF and FGF. ELISA analysis revealed high expression levels for VEGF and FGF in the human skin fibroblast line Detroit 551 after hTGF-β1 treatment (Figure 7A,B). However, in comparison with the hTGF-β1 treatment, applying RC28-E for 48 h significantly reduced VEGF and FGF levels in the cell culture medium. Moreover, the reduction of VEGF levels by RC28-C1 (VEGF-trap) was more pronounced compared to RC28-E. Furthermore, the reduction of FGF levels by RC28-C2 (FGF-trap) was consistent with RC28-E. The expression of VEGF and FGF mRNAs were detected by qRT-PCR and were significantly higher in the hTGF-β1 treatment group compared to the control group. RC28-E treatment could significantly suppress the expression of these genes, as shown in Figure 7C,D. In addition, RC28-E exhibited a higher inhibition compared to RC28-C1 (VEGF-trap) and RC28-C2 (FGF-trap) at the same concentration.

TGF-β has been reported as a crucial profibrotic cytokine that induces renal fibrosis via Smad-dependent signaling pathways. Therefore, we were interested in the functional role of RC28-E involved in the TGF-β pathway; the effects of RC28-E on TGF-β signaling pathways were examined in the human skin fibroblast line Detroit 551. As shown in Figure 7E, the TGF-β1 increased the level of phosphorylated Smad2/3, while RC28-E dramatically suppressed the phosphorylation activity of Smad2/3, which inhibited the activation of the Smad pathway. These results indicated that RC28-E inhibited hTGF-β1-mediated myofibroblast transition of human skin fibroblast line Detroit 551 by a mechanism dependent of disruption of Smad activation. We also examined the efficacy of RC28-E in the AKT signaling pathway. As shown in Figure S4, RC28-E suppressed the phosphorylation activity of AKT, and reached significance at the concentration of 2400 nM; however, the phosphorylation activity of AKT had no statistical difference with that of the hTGF-β1 induced group.

## 4. Discussion

Pulmonary fibrosis is a devastating lung disorder with incomprehensive pathogenesis and limited treatment options. Under normal conditions of lung injury, chemokines such as TGF-β1, VEGF, FGF, and PDGF are central to the processes of epithelial cell proliferation and differentiation [33]. TGF-β1 is a multifunctional protein that can, depending on the target cell, either stimulate or inhibit cell proliferation and differentiation [34,35]. Although TGF-β plays a predominant role in pulmonary fibrosis, it is not preferentially chosen as a mono therapeutic target for anti-fibrosis.

VEGF and bFGF expressions are elevated in patients with asthma, and these proteins are associated with increased vascularity [36]. In a preclinical model of pulmonary fibrosis (bleomycin (BLM)-induced pulmonary fibrosis), VEGF-A positively stained cells were in the absence of increased angiogenesis in the fibrotic area. Transfection of anti-VEGF gene therapy, in the form of the sFlt-1 gene, resulted in the attenuation of pulmonary fibrosis with a reduction of collagen deposition and additional anti-inflammatory and anti-angiogenic effects [37]. In another study, liver fibrosis resulting from chronic exposure to CCl4 was markedly decreased in liver of FGF1/FGF2-deficient mice [15]. These results suggest that FGFs, and especially FGF2, play a key role in chronic or prolonged wound healing as well as in the progression of disease accompanied by fibrosis. The present study testing RC28-E, previously identified as a block of VEGF-A, PlGF, FGF-2, and PDGF, demonstrated that RC28-E attenuates established lung fibrosis by blocking VEGF and FGF-2 in an in vivo bleomycin-induced model.

Inhibition of the pathway regulated by VEGF, FGF, CTGF, PDGF, and TGF-β has been suggested to provide novel therapeutic approaches for the treatment of fibrosis associated with chronic lung diseases. Furthermore, we found that TGF-β promotes the expression of FGF-2 in endothelial cells, which up-regulates VEGF synthesis [38,39,40]. As mentioned previously, each of these growth factors induced by TGF-β has a unique role in the pathophysiology of fibrosis. TGF-β is a pleiotropic mediator, and many reports indicate that direct inhibition of TGF-β leads to many side effects [41]. In this study, we showed that RC28-E was able to block TGF-β-mediated differentiation of fibroblasts in the absence of inhibition of the TGF-β receptor kinases. These data suggest that fibroblasts transform to myofibroblasts by the action of TGF-β via downstream factors that are inhibited by RC28-E. Since fibroblast transformation into myofibroblasts has been identified as a key factor of fibrosis in many organs, RC28-E may be useful in several fibrotic diseases.

The molecular processes driving fibrosis are wide-ranging and complex. TGF-β plays a major role in fibrosis, through the binding of a ligand to a serine–threonine kinase type II receptor that recruits and phosphorylates a type I receptor. This type I receptor subsequently phosphorylates Smads, which function as downstream effectors typically by modulating target gene expression [42]. Many studies have shown that targeting Smad3 can improve the development and progression of tissue fibrosis in vivo, including experimental models of renal fibrosis, pulmonary fibrosis, and liver fibrosis [43,44,45,46]. These studies showed that profibrotic TGF-β responses require the cooperative action of PDGF and ErbB receptor tyrosine kinases [47]. Moreover, the action of TGF-β is partly mediated by production of connective tissue growth factor (CTGF) and FGF-2 [48,49]. These reports demonstrate that the profibrotic effects of TGF-β are mediated by several growth factors, including PDGF, VEGF, CTGF, and FGF-2, indicating that the role of TGF-β in lung fibrogenesis may have been overestimated in previous studies. Our current results showed that RC28-E significantly reduced the VEGF and bFGF levels in TGF-β-treated fibroblasts and caused significant inhibition of phosphorylated Smad2/3. After VEGF-A combined with its receptor VEGFR-2 or NRP-1 and FGF combined with its receptor FGFR, downstream molecules were then phosphorylated and activated through phosphatidylinositide 3-kinases (PI3K) and AKT, which regulate the protein synthesis of angiogenesis and promote endothelial cell proliferation [50,51,52]. We also found that RC28-E may act by blocking VEGF/FGF-2 and its downstream AKT signaling pathway to regulate fibrosis. VEGF, FGF, and TGF-β signaling pathways acts uniquely to promote fibrosis, and inhibition of the cross-talk between VEGF, FGF, and TGF-β can attenuate fibrosis.

## 5. Conclusions

In this study, we suggest that RC28-E might alleviate lung fibrosis by neutralizing or blocking VEGF and FGF-2, thus attenuating their effects on fibroblast activation. RC28-E might be suitable as a potential agent for pulmonary fibrosis therapy.

## Supplementary Materials

The following are available online at https://www.mdpi.com/2218-273X/9/11/644/s1, Figure S1: The effects of RC28-E and RC28-C in control animals. Figure S2: Identified fibroblasts by FSP1 (fibroblast specific protein 1) and EpCAM (epithelial cell adhesion molecule) using flow cytometry, Figure S3: Effect of RC28-E on TGF-β1-induced myofibroblast marker expression in primary mouse fibroblasts, Figure S4: RC28-E inhibited the phosphorylation activity of the downstream signaling pathway molecules AKT.
