# Supplementary material for: Pharmacology Study of the Multiple Angiogenesis Inhibitor RC28-E on Anti-Fibrosis in a Chemically Induced Lung Injury Model
Source: Biomolecules. 2019 Oct 24;9(11):644. doi: 10.3390/biom9110644 (PMC6920960; doi:10.3390/biom9110644)
Supplement: Supplementary file 1 [file biomolecules-09-00644-s001.pdf]

Supplementary Materials

Pharmacology Study of the Multiple Angiogenesis Inhibitor RC28-E on Anti-Fibrosis in a Chemically Induced Lung Injury Model

Xiangying Kou [1],Yeying Sun [1], Shenjun Li [2], Weihua Bian [1], Zhihao Liu [2], Daolai Zhang [1] and Jing Jiang [1,*]

[1] Department of Pharmacology, Binzhou Medical University, Yantai 256603, Shandong, China; xiangyingkou@sina.com

[2] RemeGen Co.,Ltd., Yantai 264006, Shandong, China; lisj@remegen.cn (S.L.); liuzhihao@remegen.cn (Z.L.)

* Correspondence: jjiang@bzmc.edu.cn; Tel: +86-0535-6913053; Fax: +86-0535-6913053

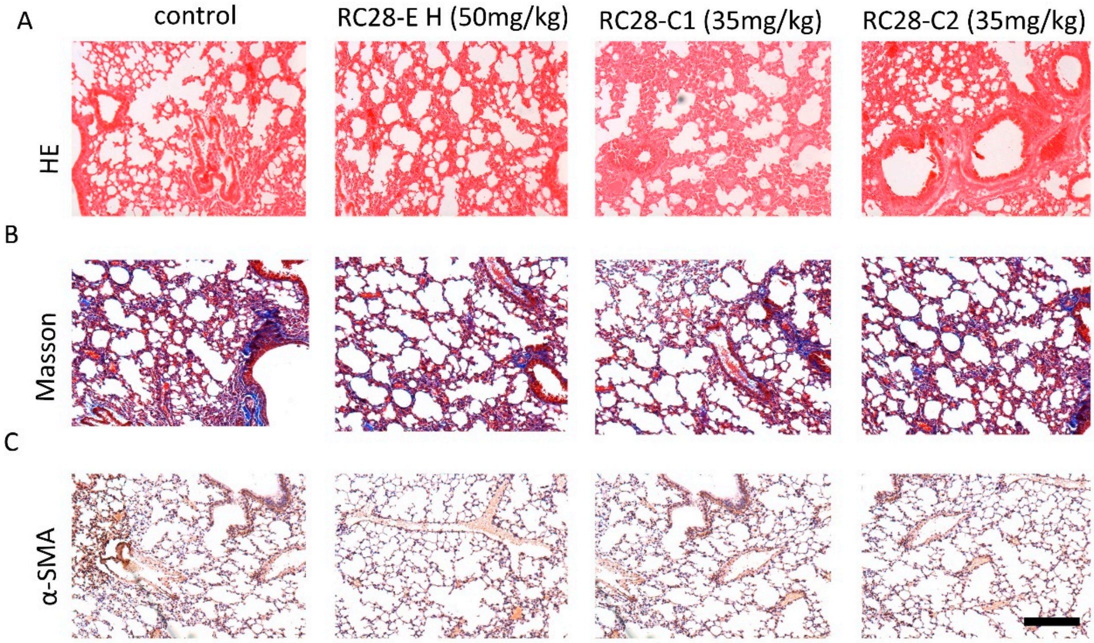

Figure S1. The effects of RC28-E and RC28-C in control animals. (**A**) Hematoxylin–Eosin (HE) and (**B**) Masson trichrome staining of normal mice lung after treatment with RC28-E, RC28-C1, and RC28-C2. (**C**) The expression of α-SMA was determined by using immunohistochemical assay after treatment with RC28-E, RC28-C1, and RC28-C2. Scale bar = 200 μm for each picture (original magnification: ×100, n = 4 per group).

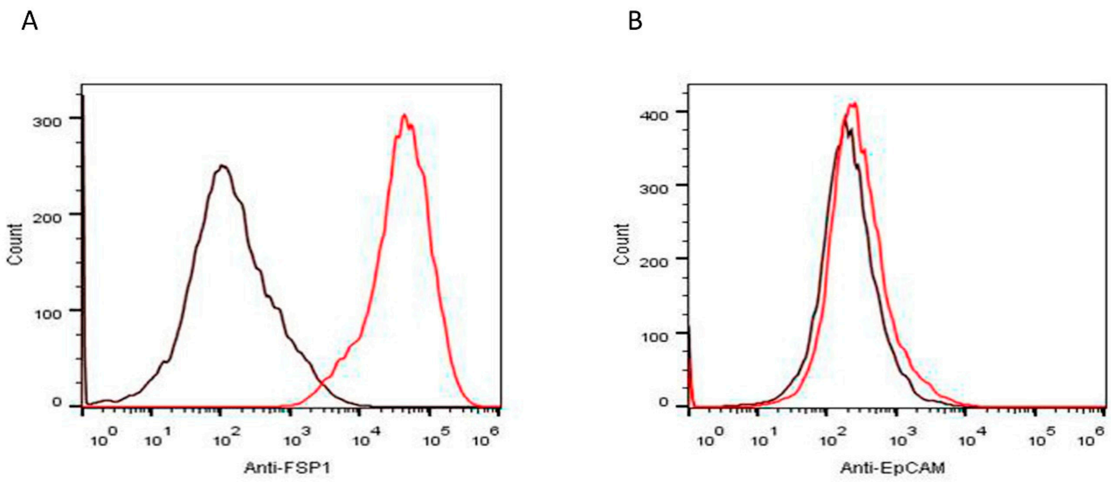

Figure S2. Identified fibroblasts by FSP1 (fibroblast specific protein 1) and EpCAM (epithelial cell adhesion molecule) using flow cytometry. (**A**) Flow cytometry analysis of isolated fibroblasts labelling FSP1with anti-FSP1 antibody (ab197896) (red). (**B**) Black—Isotype control. EpCAM was labelled with anti-EpCAM antibody (ab237385) (red). Black—Isotype control.

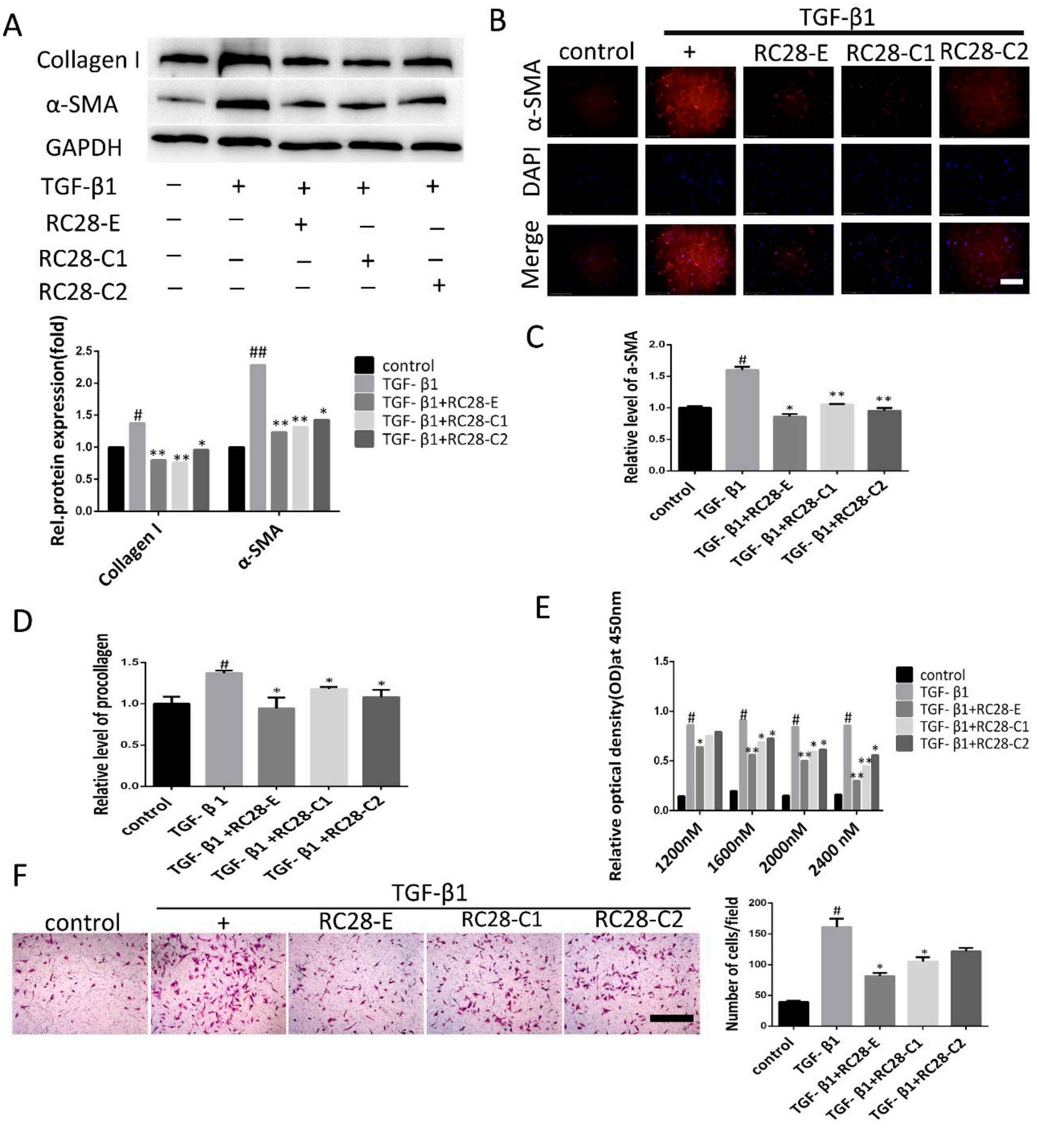

Figure S3. Effect of RC28-E on TGF-β1-induced myofibroblast marker expression in primary mouse fibroblasts. (**A**–**D**) Primary mouse fibroblasts were treated with RC28-E (2400 nM) or 2400 nM RC28-C1 (VEGF-trap) and RC28-C2 (FGF-trap) in the presence mTGF-β1 (10 ng/ml) for 48 h. (**A**) The protein expression levels of α-SMA and collagen I were examined by western blot and GAPDH as loading control. Representative gel electrophoresis bands are shown, and the expression levels of protein were quantified by densitometry and normalized to the expression of GAPDH (n = 3). Densitometry data are shown as mean ± SD. (**B**) The expression of α-SMA was detected by immunofluorescence assay. Nuclei were stained with DAPI (blue). Scale bar = 275 μm for each picture (original magnification: X200). (**C**,**D**) Changes of α-SMA and procollagen transcripts were analyzed by qRT-PCR. (**E**) Cells were treated with 1200, 1600, 2000, and 2400 nM RC28-E or 1200, 1600, 2000, and 2400 nM RC28-C1 (VEGF-trap) or 1200, 1600, 2000, and 2400 nM RC28-C2 (FGF-trap), with mTGF-β1 (10 ng/ml) as the control, for 48 h. Relative cell proliferation was determined by using cell counting kit-8. (**F**) Transwells were used for migration analysis; the migrated cells were stained with crystal violet. Results are shown as mean ± SD. *P < 0.05, **P < 0.01 versus mTGF-β1 alone. #P < 0.05, ##P < 0.05 versus control.

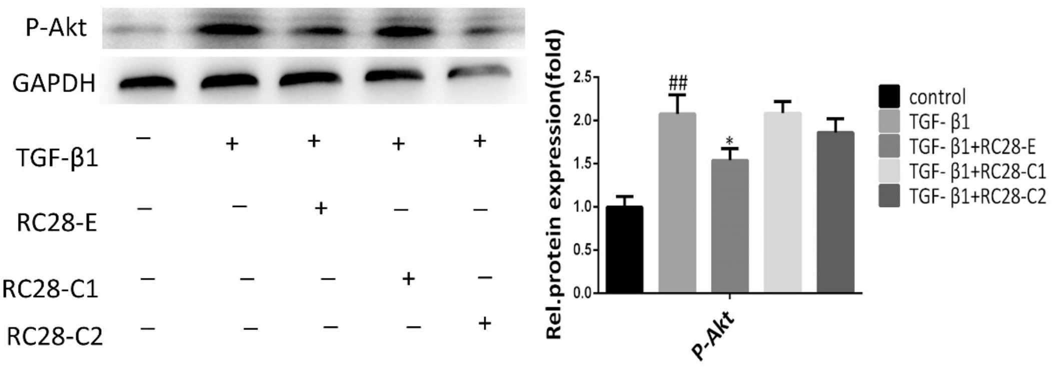

Figure S4. RC28-E inhibited the phosphorylation activity of the downstream signaling pathway molecules AKT. The protein expression levels of P-Akt were examined by western blot and GAPDH as loading control. Representative gel electrophoresis bands are shown, and the expression levels of protein were quantified by densitometry and normalized to the expression of GAPDH. Densitometry data are shown as mean ± SD. *P < 0.05, **P < 0.01 versus hTGF-β1 alone. #P < 0.05, ##P < 0.05 versus control (n = 3).
